# Severe Neurovascular and Cardiovascular Manifestations of Takayasu Arteritis: A Case Series and Review of the Literature

**DOI:** 10.7759/cureus.108590

**Published:** 2026-05-10

**Authors:** Alfakihi Ahmed Said IsmaIl, Jonathan Moyambi, Lamiaa Afendi, Meryem Haboub, Abdenasser Drighil

**Affiliations:** 1 Cardiology, Centre Hospitalier Universitaire (CHU) Ibn Rochd, Casablanca, MAR; 2 Cardiovascular Disease, Centre Hospitalier Universitaire (CHU) Ibn Rochd, Casablanca, MAR

**Keywords:** aortic insufficiency, heart failure, ischemic stroke, large-vessel vasculitis, left ventricular dysfunction, takayasu arteritis, vascular imaging

## Abstract

Takayasu arteritis is a chronic large-vessel vasculitis that predominantly affects young women and may lead to severe vascular complications. This case series is noteworthy because it describes Takayasu arteritis revealed by severe cardiovascular and neurovascular complications, including ischemic stroke and advanced heart failure, in young women without traditional cardiovascular risk factors. We report three cases of Takayasu arteritis observed at Ibn Rochd University Hospital in Casablanca in 2024. The median age was 38 years. Two patients presented with ischemic stroke, while one presented with advanced heart failure. Clinical examination revealed upper limb claudication, dyspnea on exertion, decreased or absent radial pulses, carotid bruits, and significant blood pressure asymmetry between upper limbs. Laboratory investigations showed an inflammatory syndrome with elevated C-reactive protein and erythrocyte sedimentation rate, as well as inflammatory anemia. The diagnosis was confirmed by multimodal non-invasive vascular imaging, including Doppler ultrasound, computed tomography (CT) angiography, magnetic resonance (MR) angiography, and positron emission tomography (PET) scan, fulfilling the 2022 American College of Rheumatology classification criteria. All patients received high-dose corticosteroid therapy (1 mg/kg/day) followed by progressive tapering. Standard treatment for heart failure was initiated when indicated, and anticoagulation or antiplatelet therapy was prescribed according to the presence of embolic events. Clinical and biological improvement was observed in all patients, with partial neurological recovery and a significant reduction of inflammatory markers. Takayasu arteritis may present with highly polymorphic clinical presentations, including severe cardiovascular and neurovascular complications as initial manifestations in young women without traditional risk factors. Early recognition through multimodal non-invasive vascular imaging is essential to establish the diagnosis promptly and initiate appropriate treatment, thereby reducing the risk of irreversible complications.

## Introduction

Takayasu arteritis, formerly referred to as “pulseless disease” or aortic arteritis, is a chronic large-vessel vasculitis primarily affecting the aorta and its major branches [1,2]. In the early stages of the disease, arterial wall thickening represents the hallmark pathological feature and may progressively evolve into stenosis, thrombosis, and, in some cases, aneurysm formation [2]. Disease severity is mainly related to vascular complications, including neurological, cardiac, aortic, renal, and ocular involvement [3,4]. Takayasu arteritis predominantly affects young women, most commonly between 20 and 40 years of age, with a marked female predominance [1,2]. The clinical presentation is highly heterogeneous, ranging from constitutional symptoms related to systemic inflammation to severe ischemic manifestations, which often leads to delayed diagnosis [2,3].

The diagnosis of Takayasu arteritis is based on a combination of clinical findings, inflammatory biological markers, and vascular imaging [5,6]. Laboratory investigations typically demonstrate a systemic inflammatory syndrome, including elevated C-reactive protein (CRP) and erythrocyte sedimentation rate (ESR), although no disease-specific biomarker has been identified [6]. Vascular imaging plays a pivotal role in both diagnosis and disease assessment. Non-invasive imaging modalities, such as Doppler ultrasound, computed tomography (CT) angiography, magnetic resonance (MR) angiography, and positron emission tomography (PET), allow early detection of arterial wall inflammation and luminal abnormalities [7,8]. In this study, we report three cases of Takayasu arteritis illustrating the heterogeneity of clinical presentations, the central role of multimodal imaging, and the associated diagnostic and therapeutic challenges.

## Case presentation

Case 1

A 35-year-old woman with a history of Gougerot-Sjögren syndrome presented with acute neurological deficits characterized by left hemiplegia with facial involvement and dysarthria, consistent with an ischemic stroke in the right middle cerebral artery territory. She also reported progressive fatigue, photosensitivity, and left upper limb claudication for one year. On clinical examination, absent right brachial and radial pulses were noted, associated with significant blood pressure asymmetry between the upper limbs. A systolic-diastolic carotid bruit was present. Laboratory investigations revealed a marked inflammatory syndrome, with CRP at 110 mg/L (reference range < 5 mg/L), ESR at 89 mm/hour (reference range < 20 mm/hour), and anemia of chronic disease with hemoglobin at 9 g/dL (reference range 12-16 g/dL). Serum ferritin was 315 µg/L, supporting this diagnosis in the context of systemic inflammation. Brain imaging confirmed an ischemic stroke in the right middle cerebral artery territory. CT angiography and MR angiography demonstrated multiple arterial lesions involving the carotid arteries (Figures 1, 2). Doppler ultrasound of the supra-aortic vessels showed circumferential hypoechoic wall thickening of the carotid arteries (“macaroni sign”), consistent with inflammatory involvement (Figure 3). High-dose corticosteroid therapy (1 mg/kg/day) was initiated, along with antiplatelet therapy. The clinical course was favorable, with partial neurological recovery.

**Figure 1:**
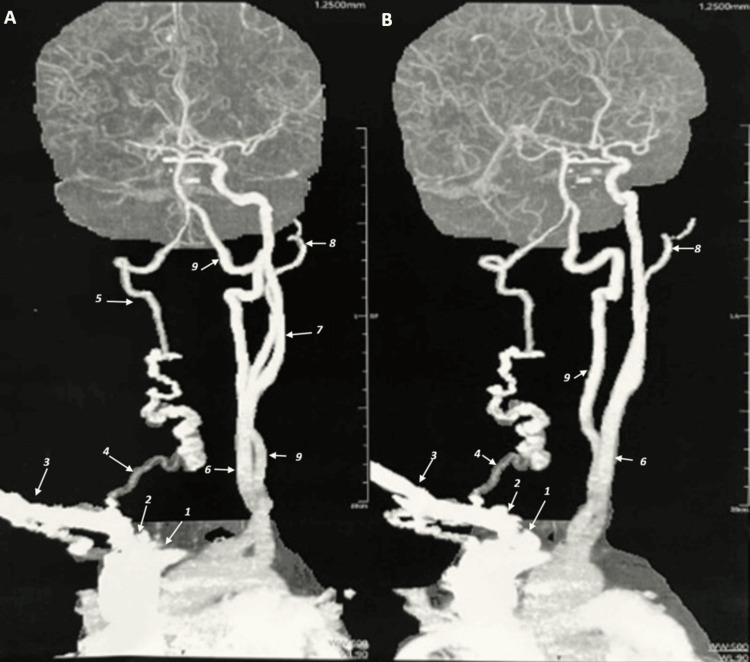
(A, B) Case 1: magnetic resonance angiography demonstrating occlusion of the brachiocephalic artery, the right common and internal carotid arteries, the ipsilateral subclavian and axillary arteries, and the V1 segment of the right vertebral artery. 1: occlusion of the brachiocephalic artery; 2: complete occlusion of the right common carotid artery; 3: right subclavian; 4: stenosis of the V1 segment of the right vertebral artery; 5: right vertebral artery; 6: left common carotid artery; 7: left internal carotid artery; 8: left external carotid artery; 9: left vertebral artery

**Figure 2:**
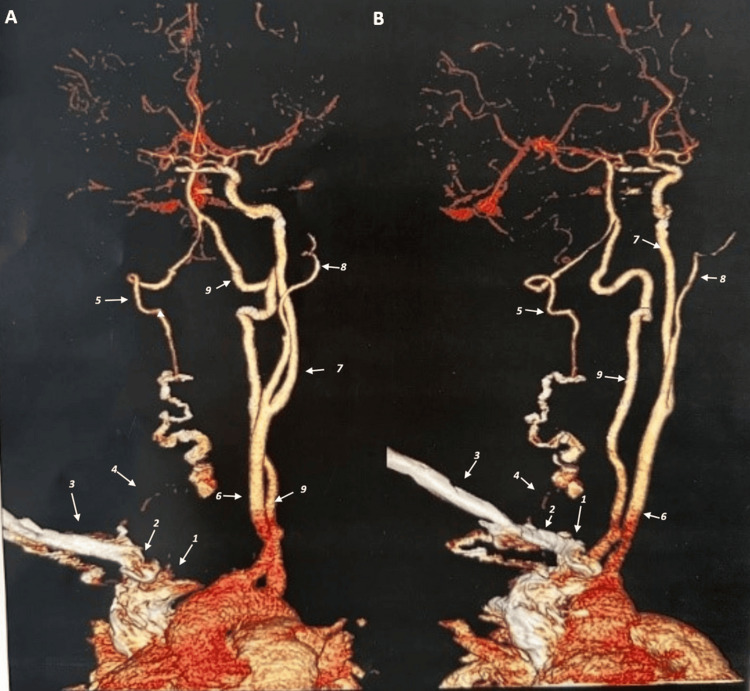
(A, B) Case 1: computed tomography (CT) angiography demonstrating occlusion of the brachiocephalic artery, the right common and internal carotid arteries, the ipsilateral subclavian and axillary arteries, and the V1 segment of the right vertebral artery. 1: occlusion of the brachiocephalic artery; 2: complete occlusion of the right common carotid artery; 3: right subclavian; 4: stenosis of the V1 segment of the right vertebral artery; 5: right vertebral artery; 6: left common carotid artery; 7: left internal carotid artery; 8: left external carotid artery; 9: left vertebral artery

**Figure 3:**
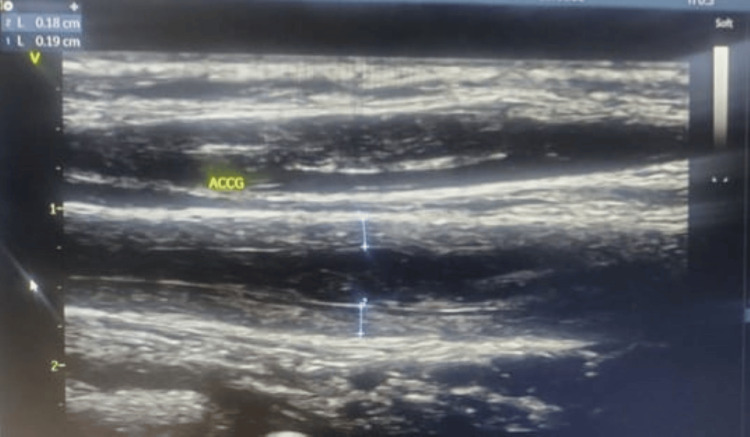
Case 1: Doppler ultrasound of the left common carotid artery showing circumferential, homogeneous wall thickening with a hypoechoic appearance (“macaroni sign”), characteristic of Takayasu arteritis. The arterial lumen is markedly narrowed, with reduced or absent flow, consistent with severe inflammatory involvement.

Case 2

A 36-year-old woman with a history of chronic migraine presented with acute neurological symptoms characterized by left hemiparesis and dysarthria, suggestive of ischemic stroke. She also reported progressive fatigue and right upper limb claudication over several months. On clinical examination, there was a decreased right radial pulse associated with significant blood pressure asymmetry between the upper limbs. A carotid bruit was detected. Laboratory investigations revealed marked inflammatory syndrome with CRP at 150 mg/L (reference range < 5 mg/L), ESR at 76 mm/hour (reference range < 20 mm/hour), and severe inflammatory anemia with hemoglobin at 7 g/dL (reference range 12-16 g/dL); serum ferritin was 367 µg/L. Doppler ultrasound of the supra-aortic vessels demonstrated circumferential hypoechoic wall thickening of the carotid arteries (“macaroni sign”), indicative of active inflammatory involvement (Figure 4). Vascular imaging using CT angiography and MR angiography revealed extensive and predominant involvement of the carotid arteries, with complete occlusion of the right internal carotid artery. As illustrated in Figure 5, imaging demonstrated severe carotid involvement, consistent with advanced-stage Takayasu arteritis.

**Figure 4:**
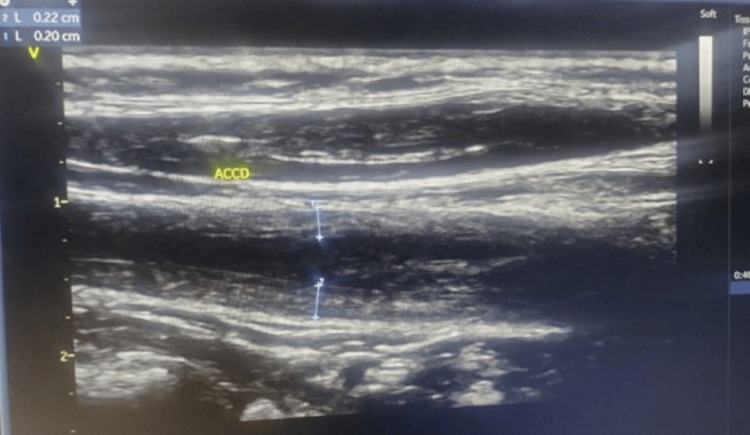
Case 2: Doppler ultrasound of the supra-aortic vessels demonstrating circumferential hypoechoic wall thickening of the right common carotid artery (“macaroni sign”), consistent with inflammatory involvement in Takayasu arteritis.

**Figure 5:**
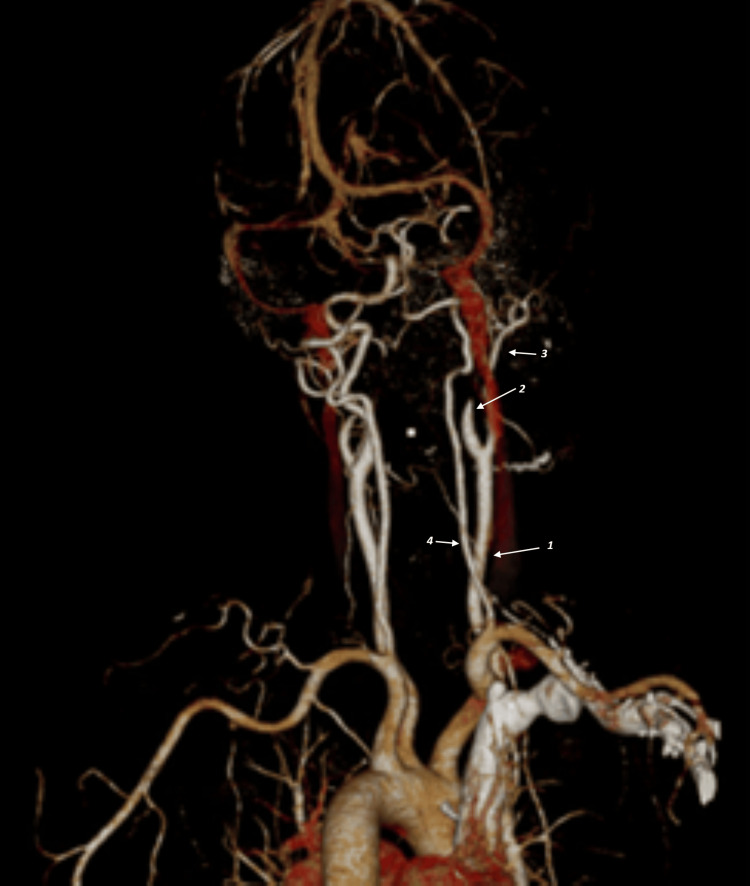
Three-dimensional computed tomography (CT) angiography of the supra-aortic vessels demonstrating complete occlusion of the right internal carotid artery and moderate wall thickening of the common carotid artery, consistent with advanced Takayasu arteritis. 1: right common carotid artery; 2: occlusion of the right internal carotid artery; 3: right external carotid artery; 4: right vertebral artery

Brain MRI confirmed an acute ischemic stroke in the right middle cerebral artery territory. A 12 mm intraluminal thrombus was identified at the M1-M2 junction, with absence of distal branch opacification, indicating severe impairment of cerebral perfusion (Figure 6). The predominance of occlusive lesions reflects an advanced stage of the disease. The diagnosis of Takayasu arteritis was established according to the 2022 American College of Rheumatology classification criteria [5]. High-dose corticosteroid therapy (1 mg/kg/day) was initiated, combined with appropriate antithrombotic treatment. Clinical evolution was favorable, with partial neurological recovery and reduction of inflammatory markers.

**Figure 6:**
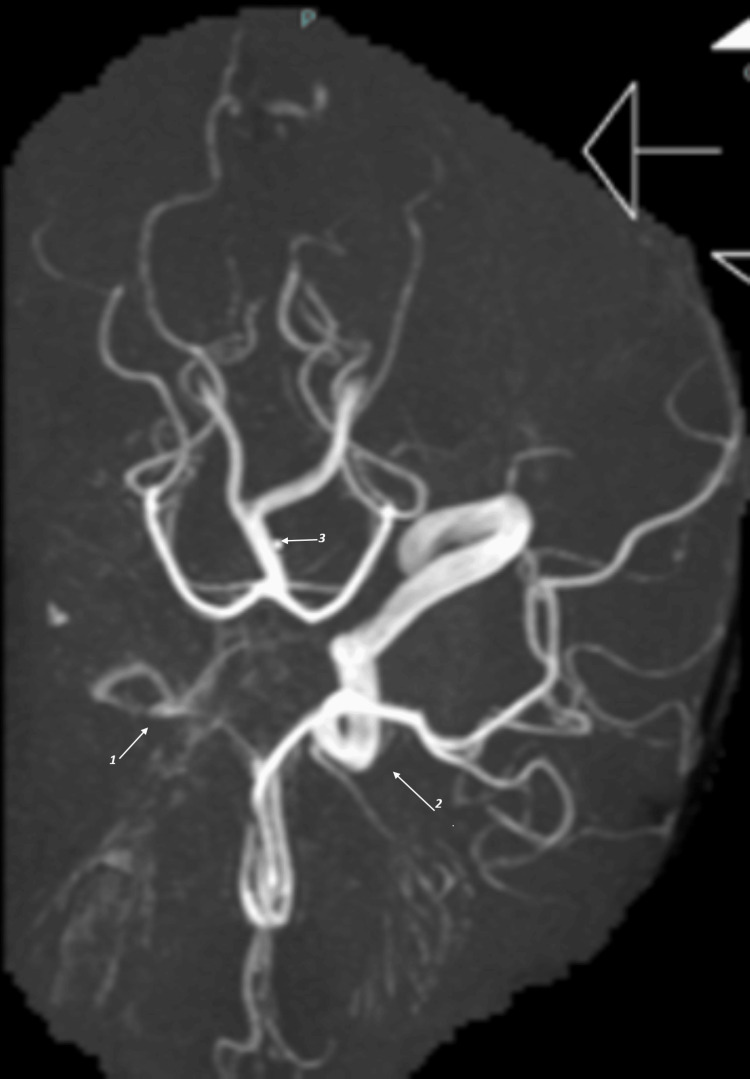
Case 2: magnetic resonance angiography of the brain demonstrating asymmetry of the circle of Willis, with suspected thrombotic occlusion at the M1-M2 junction of the right middle cerebral artery and marked reduction of distal branch opacification, indicating severe cerebral hypoperfusion. 1: thrombotic occlusion at the M1-M2 junction of the right middle cerebral artery and marked reduction of distal branch opacification; 2: left internal carotid artery; 3: basilar artery

These neuroimaging findings correlate with the upstream occlusion of the right internal carotid artery, highlighting the severity of vascular involvement.

Case 3

A 43-year-old woman presented with progressive dyspnea and signs of congestive heart failure (New York Heart Association (NYHA) class IV). Physical examination revealed pulmonary crackles, lower limb edema, and a systolic murmur. Peripheral pulses were diminished, with significant blood pressure asymmetry between the upper limbs. A carotid bruit was also present. Laboratory investigations revealed an inflammatory syndrome with CRP at 70 mg/L (reference range < 5 mg/L), ESR at 100 mm/hour (reference range < 20 mm/hour), and inflammatory anemia with hemoglobin at 10 g/dL (reference range 12-16 g/dL).

Transthoracic echocardiography demonstrated dilation of the ascending aorta associated with severe aortic regurgitation and mild left ventricular systolic dysfunction, with an estimated ejection fraction of 46%. This left ventricular dysfunction may be related to inflammatory involvement of the coronary circulation and/or to volume overload secondary to aortic regurgitation (Figures 7, 8). Cardiac and aortic CT confirmed aneurysmal dilation of the ascending aorta, consistent with the structural abnormalities observed on echocardiography. Vascular imaging also demonstrated dilation of the ascending aorta associated with stenotic lesions involving major arterial trunks (Figure 9). A PET scan revealed increased metabolic activity along the aortic wall, consistent with active inflammation (Figure 10). MR angiography suggested diffuse involvement of the aorta and its major branches, with predominant involvement of the right vertebral artery. In particular, a long-segment stenosis of the right vertebral artery involving the V3 and V4 segments was identified, consistent with inflammatory large-vessel vasculitis (Figure 11). There was no evidence of infective endocarditis, based on the absence of valvular vegetations on echocardiography, negative blood cultures, absence of an infectious syndrome, and favorable clinical evolution without antibiotic therapy.

**Figure 7:**
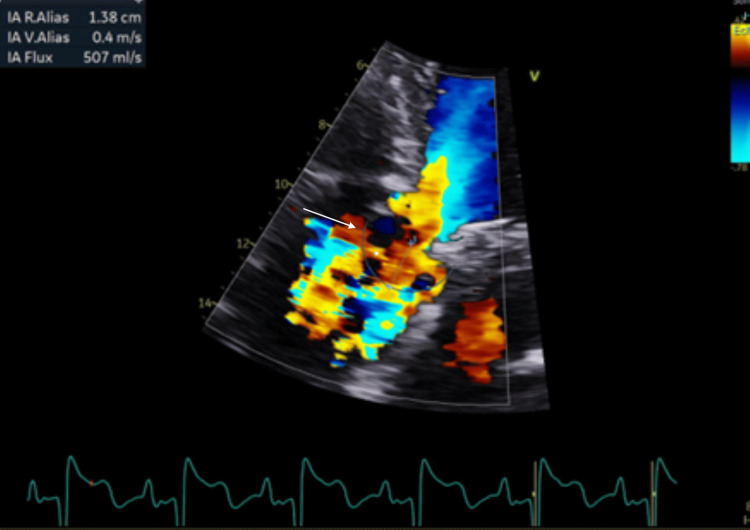
Case 3: transthoracic echocardiography demonstrating severe aortic regurgitation with a large regurgitant jet, indicating significant valvular dysfunction secondary to aortic root dilation in Takayasu arteritis.

**Figure 8:**
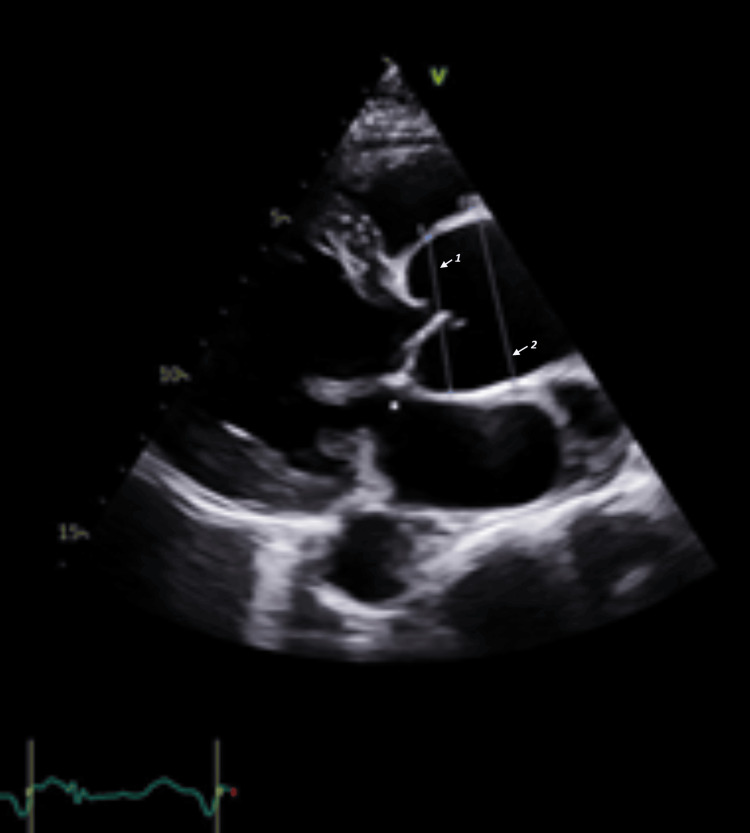
Case 3: transthoracic echocardiography in the parasternal long-axis view demonstrating marked dilation of the aortic root and ascending aorta, involving the sinuses of Valsalva and the sinotubular junction, consistent with inflammatory aortic involvement in Takayasu arteritis. 1: sinuses of Valsalva measured 43 mm in diameter; 2: sinotubular junction measured 47 mm in diameter

**Figure 9:**
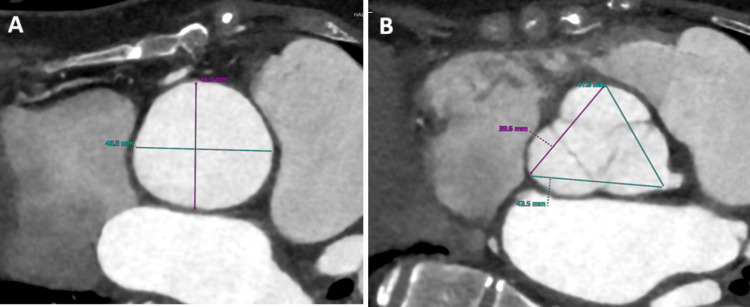
Cardiac computed tomography demonstrating dilation of the ascending aorta in Takayasu arteritis. (A) Axial image demonstrating significant dilation of the ascending aorta (maximum diameter approximately 46 mm), consistent with aneurysmal involvement. (B) Multiplanar reconstruction showing enlargement of the aortic annulus with measured dimensions. These findings explain the presence of severe aortic regurgitation in this patient.

**Figure 10:**
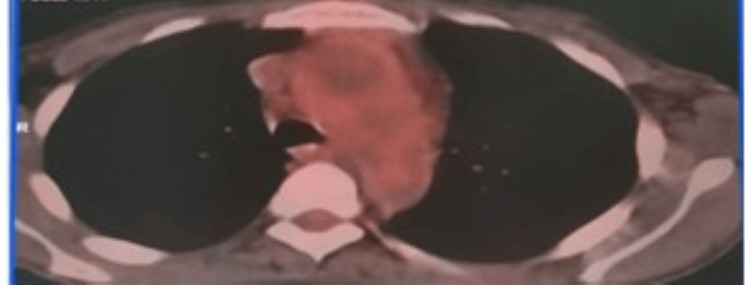
Case 3: positron emission tomography (PET) scan showing hyperactivity of the arterial wall of the aortic arch and ascending thoracic aorta. The presenting sign of Takayasu disease in this patient was severe aortic insufficiency.

**Figure 11:**
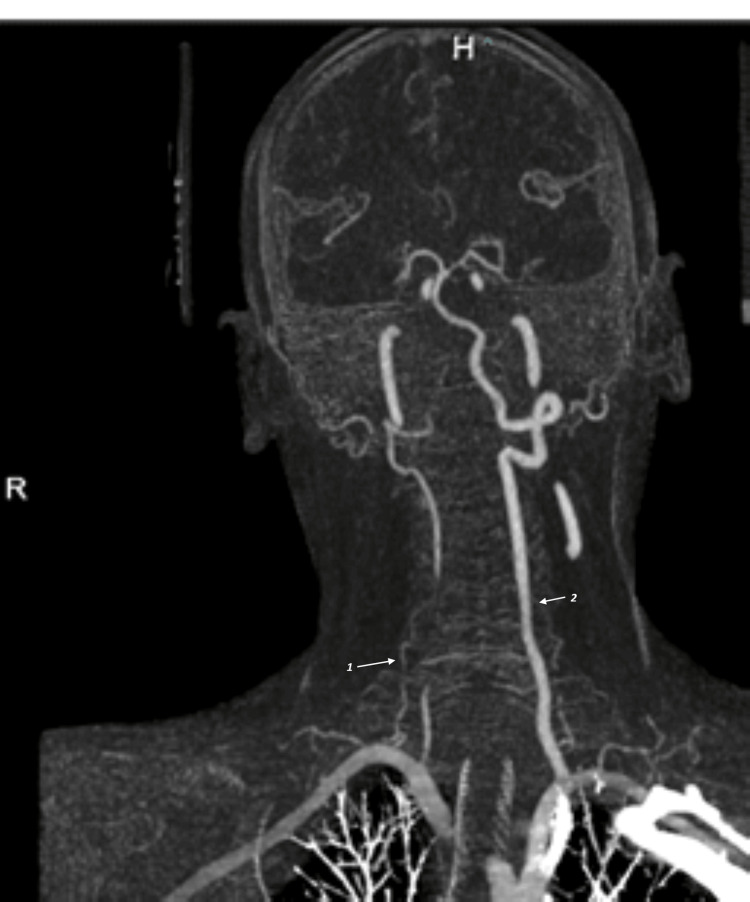
Magnetic resonance angiography demonstrating a long-segment stenosis of the right vertebral artery involving the V3 and V4 segments. This diffuse luminal narrowing is consistent with inflammatory involvement of large vessels in Takayasu arteritis. 1: right vertebral artery; 2: left vertebral artery

The diagnosis of Takayasu arteritis was established according to the 2022 American College of Rheumatology classification criteria [5]. High-dose corticosteroid therapy was initiated along with standard treatment for heart failure. The clinical course was favorable, with improvement in symptoms and reduction of inflammatory markers. After achieving adequate control of the inflammatory phase with corticosteroid therapy, the patient underwent a successful Bentall procedure. The surgery was performed during the quiescent phase of the disease to reduce the risk of complications. The postoperative course was uneventful, with marked clinical improvement.

Progressive characteristics

All patients had received corticosteroid therapy at a dose of 1mg/kg/day for one month, then dose regression, with all corticosteroid-related adjuvant measures. Treatment for heart failure was initiated in all patients. Anticoagulation was initiated in patients who had experienced embolic events, and aspirin alone was proposed in patients without embolic events.

The timeline presented in this study provides a comprehensive overview of the clinical course, diagnostic process, and therapeutic management of our patients (Table 1). It clearly illustrates the progressive and often insidious nature of Takayasu arteritis, beginning with non-specific systemic symptoms and evolving toward severe vascular complications such as ischemic stroke and advanced cardiac involvement. This temporal analysis highlights the diagnostic delay frequently encountered due to the heterogeneous clinical presentation. It also underscores the pivotal role of multimodal imaging in establishing the diagnosis and assessing disease extent. Notably, the rapid clinical and biological improvement observed following corticosteroid therapy emphasizes the importance of early recognition and timely treatment in preventing irreversible complications.

**Table 1:** Timeline of clinical presentation, diagnostic workup, and therapeutic management of patients with Takayasu arteritis. ACR: American College of Rheumatology; CT: computed tomography; MR: magnetic resonance; PET: positron emission tomography; SBP: systolic blood pressure; NYHA: New York Heart Association

Timeframe	Clinical events
≥1 year before diagnosis	Onset of upper limb claudication and photosensitivity in two patients
Months before admission	Progressive dyspnea on exertion (NYHA II-IV)
At admission	Acute ischemic stroke in two patients; advanced heart failure with severe aortic regurgitation in one patient
Initial evaluation	Decreased or absent radial pulses; carotid bruits; blood pressure asymmetry between upper limbs; inflammatory anemia
Diagnostic workup	Doppler ultrasound, CT angiography, MR angiography, and PET scan confirming large-vessel vasculitis
Diagnosis confirmed	Fulfillment of 2022 ACR classification criteria for Takayasu arteritis
Treatment initiation	High-dose corticosteroid therapy (1 mg/kg/day) started
2 weeks after treatment	Significant clinical improvement and reduction of inflammatory markers
Follow-up period	Partial neurological recovery; stabilization of cardiac function

## Discussion

Takayasu disease was first described in 1908 by Mikito Takayasu. Its clinical presentations are highly variable, depending on systemic inflammatory outbreaks and, above all, the impact of complications (complications of stenoses and aneurysms on the arterial tree). However, we do not rule out asymptomatic cases that have been discovered by chance [3].

The pathogenesis of Takayasu disease has not yet been elucidated. However, several hypotheses have been put forward: an infectious origin, notably due to its frequent association with tuberculosis; an autoimmune origin has also been suggested, due to its association with autoimmune diseases [1].

This was the case in our study, where one of the patients had already been treated for Gougerot-Sjögren syndrome. Anatomopathological examination could provide a definitive diagnosis, but arterial biopsy should be reserved for patients requiring revascularization and is no longer necessary for diagnosis. Stenoses frequently alternate with aneurysmal dilatation. Takayasu disease mainly affects women between the ages of 20 and 40 [2], as was the case with our patients, with an average age of 38.

Cardiac involvement

Takayasu arteritis can lead to various types of damage. The incidence of cardiac involvement in Takayasu disease is 40% and constitutes a criterion of severity [4]. In our study, our patients had moderately reduced left ventricular systolic function associated with disturbances in segmental kinetics, which explains the extent of inflammation in the coronary arteries. However, it is prudent to wait beyond the inflammatory phase before assessing the benefit of coronary revascularization [9,10].

Valvular damage is not unknown in the literature. Aortic insufficiency was one of the revealing features of the disease in our study. In our cardiological investigations, aortic valve disease was detected as reported by Ren et al. [11]. Valvular damage is the consequence of either annular dilatation secondary to an aortic aneurysm or retraction of the aortic cusps, or both [12].

Stroke was by far the most common predictor of Takayasu disease in our study. Several publications have made the same observation, so it would be reasonable to consider that in the event of a cerebrovascular accident in a young woman with no cardiovascular risk factors, Takayasu disease could be the first diagnosis to be evoked and ruled out [13].

The diagnosis of Takayasu disease is largely based on vascular imaging [7]. Angiography used to be the gold standard, but today, non-invasive imaging provides sufficient evidence to support the diagnosis. Doppler ultrasound is widely used and, in this study, was used to detect stenosis-type lesions, occlusions, aneurysms, and even thrombi in the brachiocephalic artery, the common carotid artery, the internal carotid artery, and the vertebral arteries, with the almost constant feature of hypoechoic circumferential thickening of the arterial wall (macaroni sign).

Angiomodensitometry and MR angiography also provide excellent analysis of the vascular lumen [11]. Millimeter-scale reconstruction of the arterial tree using these two high-performance examinations enabled us in our studies to detect occlusions and stenoses as accurately as possible and, beyond that, to make the diagnosis of ischemic stroke. PET coupled with CT (PET scan) is a useful imaging modality for detecting early lesions and active inflammation of the vessels [7].

The diagnosis of Takayasu arteritis is based on a combination of clinical, radiological, and biological evidence. Several criteria are used. However, the 2022 criteria of the American College of Rheumatology are the most commonly used.

To suspect the diagnosis of Takayasu arteritis, two conditions are necessary: age ≤ 60 years at the time of diagnosis and clear imaging evidence of large-vessel vasculitis. In addition, to confirm the disease, a minimum of 5 points must be obtained from the following criteria: female sex (+1), angina pectoris (+2), claudication of the arms and legs (+2), vascular blockage (+2), reduction in upper limb pulses (+2), abnormality of the carotid artery (+2), systolic blood pressure difference in the upper limbs ≥ 20 mmHg (+1), number of arterial territories affected (+1 to +3 ), symmetrical involvement of an even artery (+1), and involvement of the abdominal aorta with renal or mesenteric involvement (+3) [5]. Finally, a patient can be classified as having Takayasu arteritis if, in addition to the conditions required above, he or she has a score ≥ 5 points. In our study, all patients fulfilled all the criteria required to confirm the diagnosis of Takayasu disease.

Biology does not include any specific test for Takayasu disease; its only interest is to authenticate an inflammatory syndrome [6]. In our series of cases, all the patients presented a well-established inflammatory syndrome predominated by elevated CRP and ESR and the systematic presence of an anemia of chronic disease. This biological inflammatory syndrome was used to assess the response to corticosteroid therapy, which was spectacular after two weeks. Anticoagulation was initiated in patients who had experienced embolic events, and aspirin alone was proposed in patients without embolic events [14]. The neurological deficit in both patients had partially recovered. Recent advances in the management of Takayasu arteritis have emphasized the importance of cardiovascular risk modification and the use of emerging targeted therapies, particularly in patients with severe vascular involvement, as highlighted by Armstrong et al. [15].

## Conclusions

Takayasu arteritis remains a rare but potentially life-threatening large-vessel vasculitis characterized by highly heterogeneous clinical presentations, often leading to diagnostic delay and increased risk of irreversible vascular complications. Our case series illustrates the wide clinical spectrum of the disease, including ischemic stroke and severe cardiac involvement, and underscores the pivotal role of multimodal imaging in both diagnosis and disease assessment. Early diagnosis, guided by inflammatory markers and advanced imaging techniques, is crucial for the timely initiation of immunosuppressive therapy and for improving clinical outcomes. Particular attention should be paid to the early recognition of warning signs in young patients, especially women, such as unexplained fatigue, pulse asymmetry, and significant blood pressure differences between limbs. Early identification of these features may allow diagnosis at a pre-complication stage and help prevent severe vascular damage, given that the disease often responds favorably to corticosteroid therapy. These findings highlight the need to systematically consider Takayasu arteritis in young patients presenting with atypical vascular, neurological, or cardiac manifestations.
